# Derivation of occupational exposure levels (OELs) of Low-toxicity isometric biopersistent particles: how can the kinetic lung overload paradigm be used for improved inhalation toxicity study design and OEL-derivation?

**DOI:** 10.1186/s12989-014-0072-2

**Published:** 2014-12-20

**Authors:** Jürgen Pauluhn

**Affiliations:** Global Drug Discovery, Bayer HealthCare, Bayer Pharma AG, Toxicology, Wuppertal, D-42096 Germany; Hannover Medical School, Hannover, Germany

**Keywords:** Nano-size particles, Micronsized particles, Repeated inhalation exposure, Disposition, Dissolution, Volume displacement lung overload

## Abstract

**Background:**

Convincing evidence suggests that poorly soluble low-toxicity particles (PSP) exert two unifying major modes of action (MoA), in which one appears to be deposition-related acute, whilst the other is retention-related and occurs with particle accumulation in the lung and associated persistent inflammation. Either MoA has its study- and cumulative dose-specific adverse outcome and metric. Modeling procedures were applied to better understand as to which extent protocol variables may predetermine any specific outcome of study. The results from modeled and empirical studies served as basis to derive OELs from modeled and empirically confirmed directions.

**Results:**

This analysis demonstrates that the accumulated retained particle displacement volume was the most prominent unifying denominator linking the pulmonary retained volumetric particle dose to inflammogenicity and toxicity. However, conventional study design may not always be appropriate to unequivocally discriminate the surface thermodynamics-related acute adversity from the cumulative retention volume-related chronic adversity. Thus, in the absence of kinetically designed studies, it may become increasingly challenging to differentiate substance-specific deposition-related acute effects from the more chronic retained cumulative dose-related effects.

**Conclusion:**

It is concluded that the degree of dissolution of particles in the pulmonary environment seems to be generally underestimated with the possibility to attribute to toxicity due to decreased particle size and associated changes in thermodynamics and kinetics of dissolution. Accordingly, acute deposition-related outcomes become an important secondary variable within the pulmonary microenvironment. In turn, lung-overload related chronic adversities seem to be better described by the particle volume metric. This analysis supports the concept that ‘self-validating’, hypothesis-based computational study design delivers the highest level of unifying information required for the risk characterization of PSP. In demonstrating that the PSP under consideration is truly following the generic PSP-paradigm, this higher level of mechanistic information reduces the potential uncertainty involved with OEL derivation.

## Background

This paper focuses on the minimal prerequisites for deriving an occupational exposure limit value (OEL) of materials subsumed under the hypernym “poorly soluble low-toxicity granular particles (PSP)” at early stages of product development with yet limited human exposure data. At this stage, OELs have to be derived on the basis of regulatory-driven toxicity studies in general and repeated inhalation toxicity studies in particular. These animal studies are designed to reveal an undisputable toxicological threshold dose of adversity to occur to inhaled particles (no-adverse-effect level, NOAEL). This enables, for instance, the derivation of a no-effect level (DNEL) in humans or the setting of an OEL [1-3]. A novel simulation approach was developed to predict both NOAELs and DNELs/OELs at data lean situations and for designing repeated inhalation studies on rats to verify early predictions [4-6]. Direct extrapolation of the effect levels from animals to humans, that is to use a cumulative assessment factor (AF) of 1, has been suggested [4,7]. In this regulatory context, harmonized OECD testing guidelines [8-10] should be observed to design and execute the regulatory set of studies to fulfill the substances’ requirement for Registration, Evaluation, Authorisation of Chemicals (REACH) and Globally Harmonised System (GHS) of Classification and Labeling [2,3,7,11]. It is beyond the scope of this concise paper to reproduce the specific terminology and background detailed in these guidelines or those related to the concepts to calculate the human equivalent concentration (HEC) [12] which served as basis for the translation of data from rat inhalation studies to humans.

Knowledge about the mechanisms underlying the observed adverse key effect as well as the variables determining fate, either linked to the time-course changes of the key effect and/or associated dose at the target organ level, are basic prerequisites to define the metrics to be used for describing any cumulative dose-effect/response relationship. There is a general consensus that testing approaches should be designed to enable and advance computational tools with a particular emphasis on physiologically based pharmaco(toxico)kinetic modeling (PBPK). A key to this is linking the particle properties to both disposition/fate, and toxic effects [13]. The approach delineated in this review aimed to amalgamate effect-based “associative pulmonary particokinetics” with the conventional mass-based kinetics of deposited and retained particles in the lung. Pursuant to these paradigms, toxicity testing strategies for PSP should be designed and executed in a manner in which the incremental intermittent dosing and recovery periods match the kinetics of accumulation and clearance at the portal of entry and site of manifestation of toxicity. In following a hypothesis-based paradigm in study design, adversities related to any generic ‘kinetic lung overload’ and/or particle-specific toxicities can readily be identified as detailed elsewhere [4]. The novelty of the model constructed is the focus to interrelate the kinetic changes within the pool of phagocytes relative to the changed retained PSP volume. Past approaches addressing this issue focused on the alveolar mobility kinetics in relation to cellular PSP overload to better understand high-dose outcomes of long-term inhalation studies [14-21]. In this context, the term ‘displacement volume’ was used to define the volume of assembled structures consumed within the pool of alveolar phagocytes. Accordingly, the apparent density (ρ) of the assembled structure of PSP becomes an important critical qualifier of overload-dependent pulmonary toxicity. The basic hypothesis of the novel approach described in this paper defines any increase in the basal pool size of phagocytes above 6% to be the first step into adversity. The validation of this approach was shown to be simple and straightforward, namely increase in total cell counts retrieved in bronchoalveolar lavage (BAL) is taken as evidence to have attained the transition from homeostasis to adversity [4-6,22].

The uncertainty involved in the translational process of extrapolation and adjustment of findings from regulatory-compliant repeated exposure inhalation bioassays on rats to workplace exposures can be reduced when predicting and verifying that level of cumulative dose that prevents adversity to occur in the lung [4]. Suffice it to say, a PSP deposited and retained at the alveolar level may initiate some homeostatic change, e.g., attraction of phagocytes for particle removal. PBPK-modeling is conducive the better discriminate adversities originating from kinetic processes, e.g., kinetic particle overload, or from intrinsic particle toxicity. When adjusting the dosimetric variables typical of healthy workers, the dosimetrically adjusted human-equivalent daily exposure dose (OEL) can readily be estimated by following the principles of the HEC approach [4,5,12].

Regulatory toxicology is involved with the characterization of a ‘key event’ as an important aspect of toxicity leading to many final endpoints. This paper focuses on dosimetric issues of the key Mode of Action (MoA) resulting in kinetic lung overload. This ‘key event’ is defined as the first etiopathologic manifestation from the transition of homeostasis towards adversity and seems to be indicated best by an early increase of the pool-size of alveolar phagocytes (macrophages, AM) and neutrophilic granulocytes (PMN). These endpoints can be modeled assuming that 6% of the AM-volume can be consumed by PSPs without changes in pathways causing an increased recruitment of phagocytes or inflammatory cells, i.e., processes that precede additional adversities (for details see [15]). The validity of modeled outcomes with focus on the degree of overload-related increased pool-size of phagocytes and proportionally decreased elimination kinetics of particles, was cross-validated by empirical data [4,6,23] and served as cornerstone to support the approach taken. The adversities occurring beyond this overload-threshold may follow multiple and complex cumulative-dose-dependent Adverse Outcome Pathways (AOP). Such an AOP is a conceptual construct that portrays existing knowledge concerning the pathway of causal linkages between a molecular initiating event and a final adverse effect at a biological level of organization that was proposed as relevant for regulatory decisions [24]. The adverse effect occurring with PSP require homeostasis or adaptive responses to be exceeded. However, in this particular case, the issue of dosimetry and kinetics is given preference to the possible myriads of ‘molecular events’ initiating and controlling the changes in the pool-size and associated cellular and/or inflammatory processes. Hence, the approach taken for setting OELs utilizes the concept that the ‘cumulative pulmonary dose’ from a modeled repeated inhalation study on rats precedes the Point of Departure (POD) for secondary adverse outcomes to occur.

### Dissolution and bioavailability

No doubt, dissolution is tightly linked to the reciprocal relationship of particle size and surface area which determines the thermodynamic phase boundary, where the physical and chemical properties of the adjacent phases change abruptly. As in any chemical reaction, surface processes involve breaking and making of bonds. This is what catalysis is all about. Mere reversible physical adsorption may occur; however, the adsorbent may immediately be lost by desorption, in the presence of substances with competing or stronger binding isotherms. Hence, the term ‘surface area-/reactivity-dependence’ often preferred by toxicologists, is a highly dynamic process depending both on the kinetic and thermodynamic of factors present in the intimate microenvironment of the particle as well as of the physical and chemical properties of the particle itself. Hence, from a modeling perspective, many surface-related factors are highly probe-of-determination dependent. The characteristics of the probes used for the physicochemical qualification of PSP may not have any resemblance to those present in the biological system. Likewise, many simplified calculations of surface area based on idealized spherical structures cannot reliably mirror the more complex tree-dimensional assembly of nano-structures and associated void spaces.

Particle dissolution is dependent on the rate of solubilization (in mass/time), the surface area of particle and its crystallinity or lack thereof in the case of amorphous solids. Critically, the dissolution rate may depend on the presence of other factors that determine the degree of undersaturation in the liquid solvent layer immediately adjacent to the solid solute particle. The term “interfacial solubility” was proposed to describe the average concentration of the boundary layer involved in the dissolution process of particles. Interfacial barrier models consider interfacial transport rather than diffusion through the layer. “Solubilization” is the kinetic process involving both dissolution and precipitation, which occur at the same time but in different ratios, and usually proceed by diffusion. The disjoining pressure of small particles is greater than that of large particles, so small particles have a higher interfacial solubility. Due to their higher differential concentration, thinner diffusion layer, and increased surface area, small particles dissolve faster than larger particles. Accordingly, a thermodynamically more stable state is attained when larger particles grow at the expense of smaller particles. Thus, even if ‘particle disintegration’ would occur it would result in a thermodynamically instable conditions [25-29].

Dissolution is the process by which a solute forms a solution in a solvent. The solute, in the case of solids, has its crystalline structure disintegrated as separate ions, atoms, and molecules form. The amount of solute in a solution is not always determined by its thermodynamic solubility, but may depend on kinetics of dissolution (or precipitation). The solubilization kinetics, as well as apparent solubility can be accelerated by complexation, e.g. metal ion binding by peptides or proteins present in the lung. Solubility is commonly expressed as a concentration (e.g. mass of solute per kg of solvent). The maximum equilibrium amount of solute that can dissolve per amount of solvent is the solubility of that solvent under a given condition. The advantage of expressing solubility in this manner is its simplicity, while the disadvantage is that it can strongly depend on the presence of other species in the specific microenvironment of the particle. Despite the advances of *in vitro* dissolution in particle qualification, the *in vivo* bioavailability of retained particles in the lung remains to be difficult to judge by *in vitro* tests. The toxicological significance of translocation and disintegration of agglomerated particles commonly ignores the complex equilibria of dissolution and precipitation processes that may occur in highly compartmentalized biological systems. For example, the solubility of any inhaled metal oxides mechanically translocated from the airways into the gastrointestinal tract may show their highest solubility in gastric fluids - due to low pH - with resultant high concentration and mucosa-to-blood gradients as the prerequisite for transport across barriers and absorption. With increasing pH following absorption, supersaturated concentrations with precipitation may ensue. Such precipitates may eventually be retrieved in the liver and spleen. Thus, caution is advised to inextricably link particles found in the extrapulmonary circulation or organs to pulmonary barrier disruption.

Many *in vitro* procedures to determine dissolution appear to be poorly suited to predict the bioavailability and impact on fate with regard to dissolution in the lung. In this context it is crucial to know whether the determined *in vitro* values characterizing ‘solubility’ or ‘dissolution’ represent a *thermodynamic equilibrium solubility* or whether they represent the values associated with a metastable condition more suggestive of a *kinetic equilibrium*. For more details on the distinction between thermodynamic and kinetic equilibrium solubility, and how one can exceed the equilibrium solubility to yield a supersaturated solution, specialized literature should be consulted [28-30]. If equilibrium is not reached, a false impression on solubility is created, leading to inaccuracy when comparing different particles. Thus, as can readily be perceived, dissolution occurs up to the saturated concentration. In case the amount of bulk material used in the assay to attain saturation is markedly increased, solubility would become less (solubilized mass relative to the mass of bulk material).

Mathematical models for the dissolution of solid particles involve accounting for the complicated changes in the surface area and/or shape which occur during dissolution. Solid particles in liquids can be modeled using Nernst-Brunner type kinetics which is an extension of the Noyes and Whitney dissolution kinetics [25-27,30]:

$$ \frac{dM}{dt}=-\frac{D}{V_mh}{S}_A\times \left({C}_S-C\right) $$

where M is the mass of solid material at a given time t, S_A_ is the area available for mass transfer, D is the diffusion coefficient of the dissolving material, V_m_ is the dissolution medium volume, h is the diffusion boundary layer thickness, C is the concentration, and Cs is the substances saturation solubility. Diffusion-controlled models were further refined for single spherical particle dissolution under sink conditions and pseudo steady-state of the kinetic release of a particles homogeneously dispersed in a matrix into a medium under perfect sink conditions [30]. However, given the assumptions used to derive these models, the prediction may not be valid over the entire range of the dissolution curves due to the marked change of solid particle properties with decreasing size due to dissolution. Similarly, polydisperse particle sizes and coated particles retained in an inflammatory milieu of the lung may add another dimension of complexity to any model. Due to the longer life-time of humans, time- and dissolution-related changes in particle properties is biased to underestimate the contribution of clearance by slow dissolution.

**Figure 1:**
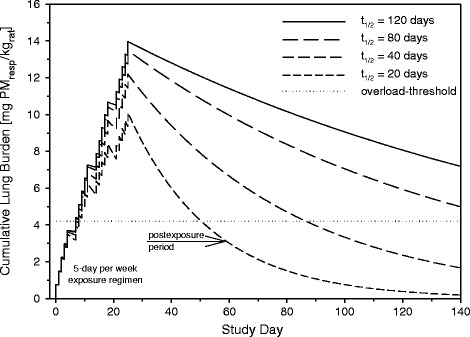
**Modeling of a 4-week repeated inhalation study in rats with minimally and/or poorly soluble particle at minimal overload conditions.** The simulations illustrate that ‘dissolution’ can readily be detected and quantified during post-exposure periods of 90-days but may remain undetected during the actual exposure period. Elimination half-times (t_1/2_) of 80 and 120 days suggest normal to beginning overload-related clearance, respectively. Half-times shorter than 60-days demonstrate a dissolution rate that might not necessarily be compatible with the PSP paradigm.

These elaborations demonstrate that any meaningful *in vivo* dissolution kinetics should use inhalation instead of instillation, insufflation or aspiration procedures to define the fate of PSP. Likewise, to make the kinetic cornerstones comparable across different substances, any dissolution kinetics should refer to the cumulative lung burdens within a meaningful range of the threshold of kinetic overload. However, as PSP may unexpectedly be more soluble in the lung than anticipated by alternative methods, pre-studies with well-rationalized cumulative lung burdens need to be executed as conceptualized in Figure 1. Typically, the dominating pathway of elimination of PSP from the lung occurs via alveolar macrophages [14-20]. At yet non-overload conditions elimination half-times of PSP retained in the lung are in the range of t_1/2_ = 60 to 90 days [4]. In case evidence of facilitated or enhanced dissolution of PSP exists a shortened half-time is observed. Once the overload-condition is attained, this is indicated by half-times exceeding t_1/2_ ~ 90 days (Figure 1). An appropriately designed and executed 1- to 4-week repeated inhalation study can serve the purpose to verify that the PSP under consideration can truly be regarded as typical PSP under *in vivo* conditions.

**Figure 2:**
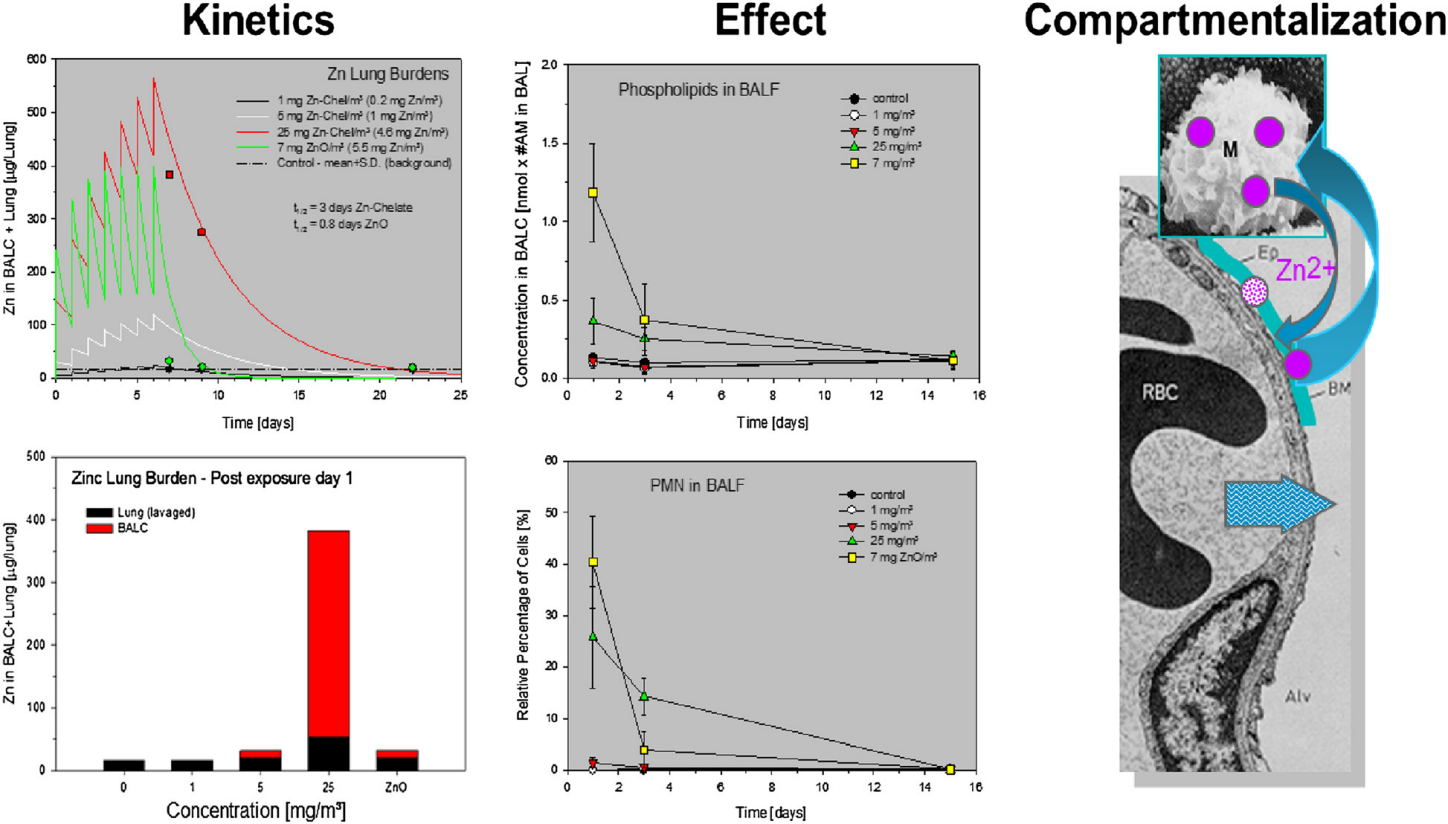
**Schematic representation outlining the interrelationship of inhaled dose and kinetics, the ensuing effect, and the sub-compartments of the lung were these dose-effect relationships are believed to occur for poorly soluble particles (Zn-chelate) at 1, 5, and 25 mg/m** ^**3**^ **and less soluble particles (ZnO) at 7 mg/m** ^**3**^ **(the last two concentrations were adjusted to attain equivalent molar concentrations of Zn).** Rats were exposed for 6 hours/day on seven consecutive days. Zn was determined in lung tissue and bronchoalveolar (BAL) cells on postexposure days 1, 3, and 15. The findings presented demonstrate that solubility may affect the compartment were dissolution and toxicity occurs. The highest toxicity occurred in ZnO-exposed rats at lower concentration of total dust (for more details see ref. [32]), which delineates the importance of dissolution rate and flux (for details see [31,32]).

The example shown in Figure 2 compares two substances commonly categorized as PSP, one is a chelate of zinc (solubility in the range of ≤1 mg/L H_2_O), the other was zinc oxide (ZnO, pigment grade; solubility in the range of ~10 mg/L H_2_O) using a 7-day 6 h/day inhalation bioassay on rats [31,32]. The mass median aerodynamic diameter (MMAD) and geometric standard deviation (GSD) were for the chelate ~1.9 μm (2.3) and for ZnO ~1.6 μm (1.6), respectively. The molar concentrations of Zn were essentially identical at 25 mg/m^3^ chelate and 7 mg/m^3^ ZnO. Empirically determined lung burdens of Zn, assisted by kinetic modeling, yielded elimination half-times of t_1/2_ = 3 and 0.8 days for the chelate and ZnO, respectively. Sub-compartmental kinetics in BAL-cells yielded appreciably different cellular doses of Zn. The chelate showed evidence of uptake by alveolar macrophages (AM) whereas the dissolution of ZnO seemed to be faster than the cellular uptake. The elimination kinetics of BAL-cells mirrored that of the total lung. Two weeks postexposure, all groups were indistinguishable from the air-exposed control. Surfactant phospholipids and neutrophils (PMN) in BAL were maximally increased following exposure to ZnO with fast clearance and reversibility of findings. A slightly slower reversibility with a shallower time course occurred in rats exposed to the chelate. Collectively, these relationships show that both particles often considered to be PSP-like dissolve rapidly in the lung. However, the dissolution rate of the chelate was slower than that of ZnO. This confers time for the chelate to be endocytosed by AM with protracted leaching from this cell. In turn, micron-sized ZnO seemed to be instantly dissolved in the alveolar lining fluids and cleared from the lung. Other authors found that both mass and surface area were effective as metrics for the toxicity of ZnO nanoparticles (NPs) [33,34]. Hence, additional published evidence supports the concept that the particle-size-dependence of dissolution of extra- and/or intra-cellularly released zinc ions may play a key role of mediating the toxicity of sufficiently soluble particles. Therefore, the types of studies shown in Figures 1 and 2 may serve as robust basis to differentiate dissolution-related and substance-specific or typical PSP-related outcomes.

### Principles of particokinetics and -toxicity

**Figure 3:**
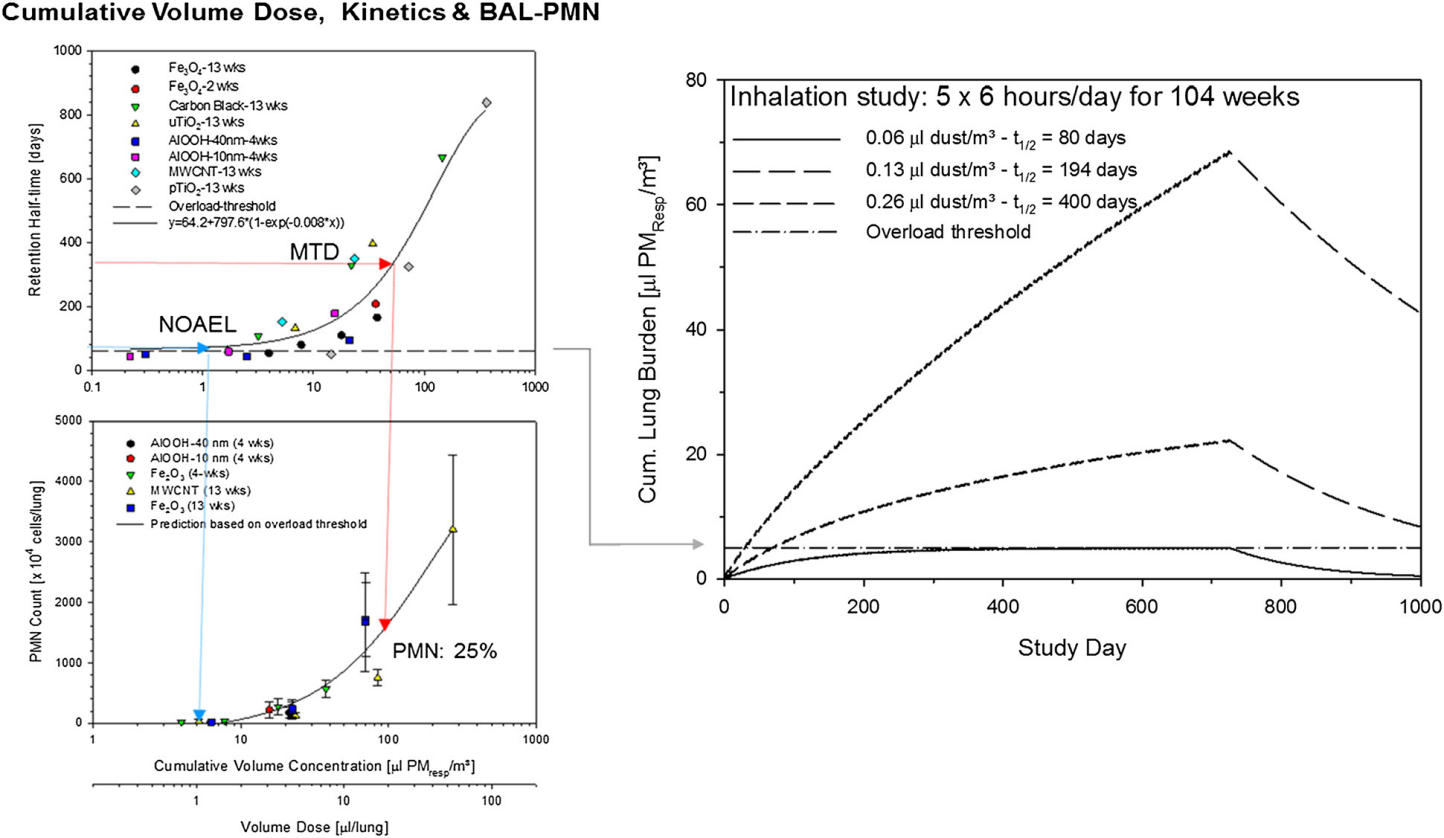
**The dependent variables ‘elimination half-time’ (top left) and ‘influx of neutrophils (PMN) in BAL’ (bottom left) were analyzed relative to the independent variable ‘cumulative dose’ expressed as C**_**respirable**_ **× Σt and both (in using an inhaled volume per exposure day this metric is proportional to the accumulated volumetric lung burden; for details see reference [** 4 **]).** The illustration supports a normal range of elimination half-times of t_1/2_ = 60 – 90 days which precedes any influx of PMN (vertical arrow left). The maximum tolerated dose (MTD) was believed to be attained at t_1/2_ = 1 year (vertical arrow right). For any chronic 2-year inhalation study on rats these targeted cornerstones are met at exposure concentrations of 0.06 μL PSP_resp_/m^3^ (NOAEL) and 0.26 PSP_resp_/m^3^ (MTD). At these cumulative lung burdens the elimination half-times are expected to be t_1/2_ = 80 and 400 days, respectively. Of note is the increasing disparity of external exposure concentrations and cumulative lung burdens at MTD: a ~4-fold difference in exposure concentrations yielded a ~17-fold higher lung burden at MTD (right) (for more details see [4]).

Kinetic modeling provides a means to interrelate the cumulative lung dose and study outcomes. These are believed to depend on two basic MoAs: (i) the MoA_I_-is believed to convey a particle-specific acute adversity whereas (ii) the MoA_II_ is attributed to endocytosed PSP following then the imposed kinetic of the phagocyte (Figure 3). Hence, from an inherent toxicity perspective, particles are expected to interact first with the lining fluids or components thereof at the site of initial deposition. The processes potentially involved have been considered in the preceding section. MoA_I_ is destined to impose acute effects caused by surfactant dysfunction either related to PSP-specific effects or adsorption phenomena [4,23]. Nonetheless acute-on-chronic aggravations cannot be ruled out following recurrent exposures. However, due to the rapid re-synthesis of acutely depleted surfactant [35], such effect-related half-times are transitional (1-3 days) relative to those mediated by MoA_II_ [36].

Unlike humans, rats appear to be more susceptible to the cumulative retention of inhaled PSP and associated pulmonary inflammation because overload-related kinetic effects occur more readily due to their smaller pool-volume of alveolar macrophages relative to humans [4,15-18,37]. With regard to PSP-related inhalation hazard characterization, missing models are a major stumbling block for providing definite regulatory guidance. Within this framework, limited guidance is given as to how ‘administered dose and dose-rate’ and/or ‘the cumulative dose’ need to be adjusted not to exceed the maximal tolerated dose (MTD) in repeated exposure studies. The physiological clearance is deemed to be exhausted at the MTD defined as elimination half-time of t_1/2_ = 1 year (Figure 3) [4]. Based on the analysis given in this illustration, an interrelationship of elimination half-time and increase of the pool of alveolar macrophages and polymorphonuclear neutrophils (PMN) can be expected. Notably, this NOAEL to MTD relationship matches a calculated volumetric lung burden of 1 to 10 μl PSP/lung. A similar relationship of a yet adaptive to an exhausted displacement volume within the pool of macrophage from 60 μm^3^ PSP/macrophage (6%) to 600 μm^3^ PSP/macrophage (60%) was postulated by Morrow [15,16]. Hence, despite the differing approaches used to estimate the transition from homeostasis to overload by Morrow [15] and Pauluhn [4] both concepts present a unifying hypothesis in defining an overload-dependent NOAEL and MTD. These concepts support the notion that the intricate physiological system of the rodents’ lung can totally be “overwhelmed” by extreme lung burdens of PSP which may deteriorate unspecifically the intricate structure and function of the lung. This calls for studies designs that utilize kinetically modeled exposure regimens to prevent excessive, and toxicologically irrelevant cumulative lung burden beyond the MTD to occur.

**Figure 4:**
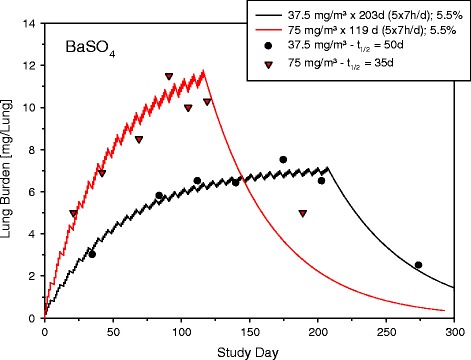
**Modeling of two C × Σt-adjusted subchronic barium sulfate inhalation studies with different particle size and surface areas from Cullen et al. [** 9 **,** 32 **].** Symbols were reproduced from figures given by these authors. The elimination half-time of BaSO_4_ in the lung of t_1/2_ ≤ 60 days as well as the fact that the equal C × Σt of the longer exposure period produced half the lung burden of the shorter exposure period suggest enhanced dissolution of particles. Fitted curves represent a 1^st-^order kinetics (for details see [4]). Opposite to the recommendation given in Figure 1, data had to be fit to the accumulation rather than the data lean elimination phase. This circumstance decreased the precision of analysis; however, elucidate further the intricate relationship of dissolution at possibly saturated conditions and MoA_II_-related clearance.

Another salt commonly presumed to be a PSP is BaSO_4_ (solubility in water ~3 mg/L H_2_O [38]). Barium sulfate was examined in rat inhalation studies as nano-sized particles (41.4 m^2^ g^−1^; BET [39]) of 1-week (postexposure period 3 weeks) [40] and as fine particles (3.1 m^2^ g^−1^; calculated) in a study of 17 and 29 weeks followed by postexposure periods of 3 months [41]. The MMAD and GSD were ~1.5 μm (2.1) and 4.3 (1.7), respectively. Following exposure to the nano-sized particles 70% of the lung burdens were cleared within a postexposure period of 3 weeks whereas the fine particles were eliminated at least twice as fast as would be expected by macrophage mediated clearance (Figure 4). The respective maximum lung burdens were about 12 mg BaSO_4_/lung and 6 mg BaSO_4_/lung [41,42]. These studies were tolerated without any evidence of pulmonary toxicity despite increased solubility and 10-fold differences in particle surface areas. This outcome supports the notion that *in vitro* “insolubility” in water cannot readily be translated to that present in the pulmonary system.

### Interdependence of metrics on dose

In the classical overload model, sixty percent of the available pool volume of phagocytes (Vd) was considered to be equal to the maximum theoretical ‘volume of distribution’ of retained PSP is the lung [15]. The tenth of this fraction is considered to be the maximum yet homeostatic Vd. For comparisons across species it was assumed that these fractions do not change with cellular size. The devised one-compartmental approach is a conservative simplification because lymphatic drainage and dissolution are neglected. However, due to the longer life-time of humans, these additional components may be conducive to non-negligible interstitial/lymphatic additional clearance [43]. Significant translocation of PSP into draining lymph nodes requires lung burdens high enough to cause overload-related inflammation [4]. Hence, kinetically modeled NOAELs are considered to be sufficiently conservative when using a one-compartmental concept with the trade-off that lung burdens at increasing overload-conditions are biased to be over-estimated due to the negligence of interstitial clearance. Thus, the model devised is implicitly over-conservative but considered to be more robust as it prevents any over-parameterization as often dictated by multi-compartmental approaches. Based on the data from diverse particle inhalation exposure studies on rats (Figure 3) as well as the deliberations of other experts [14,18-20] a 6% volumetric overload threshold within the pool of alveolar phagocytes was used for defining the transition from adaptation to adversity.

**Figure 5:**
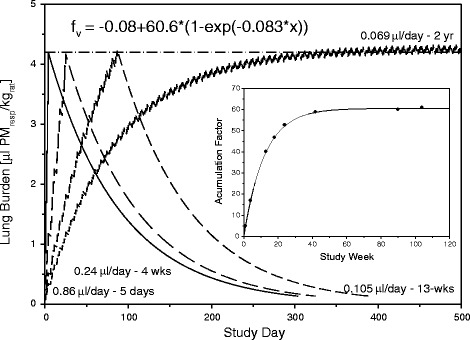
**Dependence of the exposure regimen on the predicted volumetric cumulative PSP volume at the kinetic overload threshold of 4.2 μL/kg**_**rat**_ **(dashed horizontal line; for details see** **[** 4 **]** **).** For each of the shown C × Σt –relationships, this threshold is anticipated to be the NOAEL in case MoA_I_-related findings do not increase at a higher ‘C’ and shorter ‘t’ [36]. The ratio of this PSP volume to the respective dose rate to attain overload under any given condition is used to calculate the accumulation factor f_v_ (insert) to estimate the generic retention-related (MoA_II_) NOAEL (see Figure 7).

Following the logic depicted in Figure 5, the NOAEL of any study can be estimated to be 4.2 μl PSP_resp_/kg-rat as detailed elsewhere [4]. The corresponding NOAEL to MTD equivalent pooled volume of phagocytes then becomes 4.2 and 42 μl PSP_resp_/kg-rat, respectively. Assuming unit density, mass-based, this overload-range equals 4.2-42 mg/kg-rat PSP/kg-rat. On the level of a rat macrophage this available volume is about 70-700 μm^3^/cell. Cell cultures doses are often normalized to 10^6^ cells. Under *in vitro* conditions this threshold translates to a cellular dose of 0.07-0.7 10^9^ μm^3^/10^6^ cells. With 1 μl = 10^9^ μm^3^ this means for a unit density PSP 7-70 μg/10^6^ cells. While in inhalation studies a similar dose-range commonly is delivered over 28 or 90 days, in instillation or cellular bioassays this cumulative dose is administered as single bolus. These cursory estimations show that many straightforward instillation and *in vitro* cell-based studies may utilize a dose-range beyond the volumetric threshold. This means, MoA_II_-related outcomes become readily be saturated or overwhelmed. Accordingly, such types of study design can hardly reveal any volume-dependent etiopathology and a surface area/activity-dependent metrics of toxicity can be anticipated [36].

**Figure 6:**
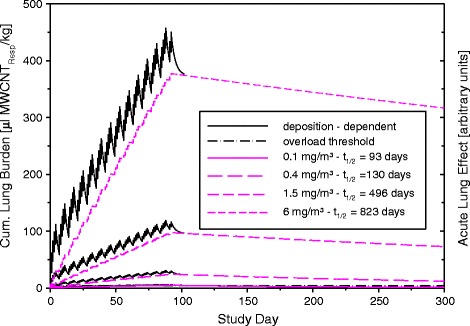
**Modeling of the accumulated volumetric lung burdens of rats exposed to respirable MWCNT (Baytubes) for 13 weeks** **[** 23 **]** **.** The deposition (MoA_I_)-dependent “effect-related kinetics” was placed into perspective relative to the retention(MoA_II_)-related kinetics following a post-exposure period of 6 months (for further details see [36]).

**Figure 7:**
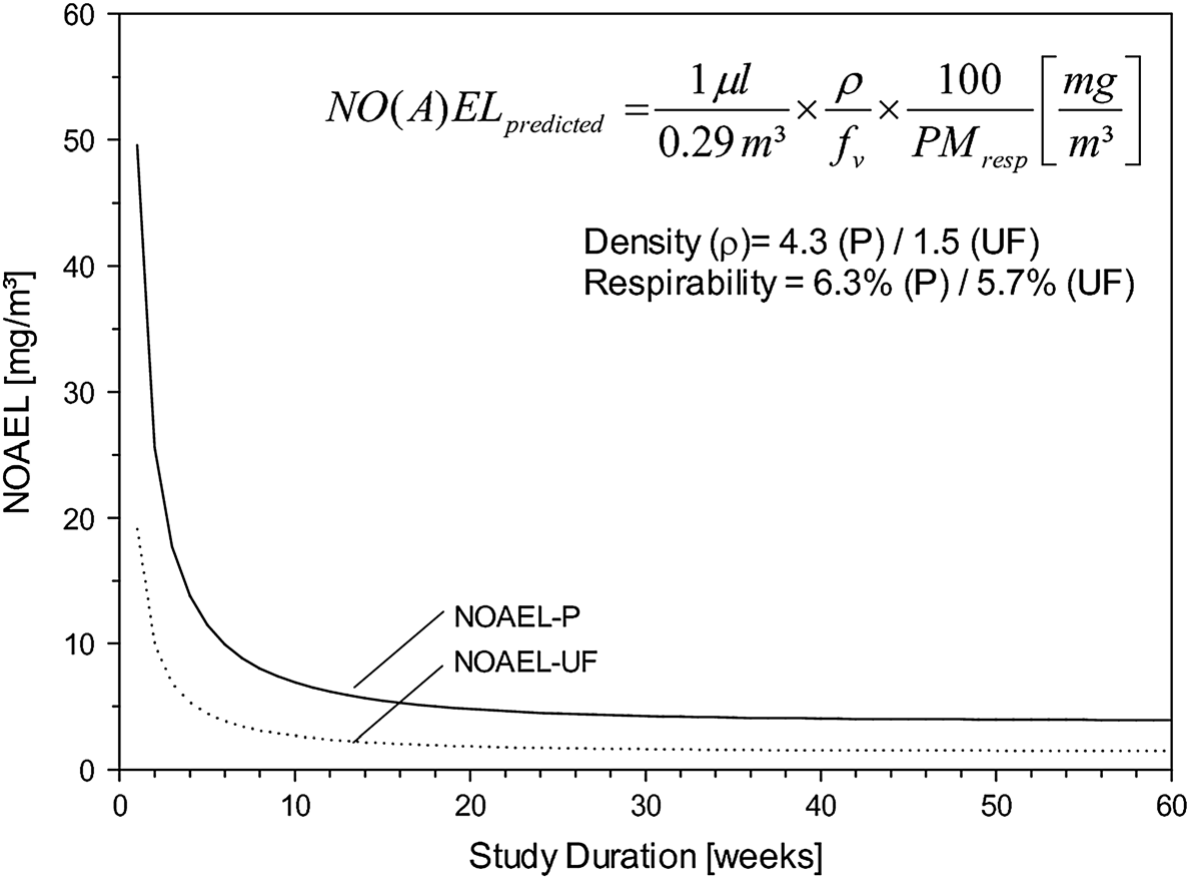
**Predicted retention-related NOAELs (MoA**_**II**_ **-based) of respirable ultrafine (UF)/pigmentary (P)-TiO**_**2**_ **at exposure durations up to 1 year (for further details see** **[** 4 **,** 36 **]** **).**

As exemplified in Figures 3 and 6, the dynamic increase in elimination half-time causes (in rats) a disproportionality of the external exposure concentration and the resultant cumulative lung burden especially following chronic inhalation. The actual exposure concentration (C) × Σexposure days (Σt) relationship needs to be constructed in a way to minimize MoA_I_-related effects relative to the MoA_II_-related effects. For risk characterization, the most predictive C × Σt-relationship is that reflecting the chronic occupational exposure regimen most, i.e., MoA_I_-related should essentially be absent at the low and intermediate levels of exposure. The predominating MoA is highly dependent on the protocol executed. High-dose short-term and low-dose long-term exposure regimens are biased to be dominated by MoA_I_ and MoA_II_, respectively (Figure 7) [36].

### Deposition-related metric (MoA_I_)

It is frequently conceived that the conceptually better alternative to particle mass as a measure of dose would be the PSP-surface thermodynamics [7]. Surface area rather than mass accounts for the fact that biopersistent particles can interact only by contact of their surface, determining an effective dose-rate by a catalytic surface reaction rate that accumulates to an effective dose with increasing residence time in the target tissue [44]. However, a finite proportion of soluble fractions of particles that may dissolve on contact with the fluids lining the airways of the lung - and so do not contribute to ‘surface area dose’ (apart from the fact of surface area-dependent facilitated dissolution [33]). Especially for less soluble particles in a C_s_-range 1 to 10 mg/L water, the dissolution flux is not only highly dependent on the physical characteristics of the particle itself, also the degree of saturation of the matrix surrounding the particle has great impact as already discussed above (Figures 1, 2 and 4). Along with these concerns, issues regarding the most appropriate unifying metric of dose are still unresolved for potentially soluble particles [45] which dissolution rates increase reciprocally with PSP-size.

### Retention-related metric (MoA_II_)

Independent of specialized subpopulations of AM, several major variables have to be accounted for as a common response to any increased endocytosis of endogenous (e.g., precipitated or denatured surfactant, pulmonary phospholipidosis, and cellular debris) and exogenous poorly soluble materials. These variables include hypertrophy (enlargement commonly caused by excessive amounts of phospholipids) of the AM with minimal, if any, increase of the pool of macrophages. To the contrary, an increase of the AM pool in the absence of any hypertrophy of cells is the most common response observed in inhalation studies with PSP [4,22]). At unequivocal overload coexistence of both may occur. Typically (in rats), at yet reasonable dose rates used in repeated inhalation toxicity studies, the pool volume of AM adapts to higher PSP load by the increased influx of cells rather than their enlargement [4,22,23].

### Modeling of the homeostatic threshold

The NOAEL of any study can readily be calculated using the 1-compartmental first-order kinetics depicted in Figure 5. A more simplified practical approach for calculating this kinetic NOAEL is given in Figure 7. The basis of this approximation is the following: the relative weight of the exsanguinated lung to body weight in rats from subchronic nose-only inhalation studies is 0.4 and 0.5% for male and female rats (Wistar-based), respectively. The first term in the equation shown in Figure 7 defines the volumetric overload threshold dose per exposure day normalized to kg-rat which was calculated to be 4.2 × μL phagocyte-pool volume/kg_rat_ [4] per ~4.5 g lung/kg_rat_ which is rounded to ~1 μL AM-pool volume/lung inhaling 0.29 m^3^/kg_rat_ per 6-hour exposure day [4]. Opposite to the extrapulmonary organs dosed by perfusion, lung burdens have to be linked to body-weight-adjusted ventilation and respirability of particles. In order to retain this link, lung burdens were expressed ‘total lung-based’ relative to the body-weight-adjusted ventilation. *f*_*v*_ = -0.08 + 60.6 (1-exp(-0.083x)) is the fractional volumetric daily exposure dose required to attain steady state as given in Figure 5 (insert). This mathematical relationship is based on an exposure regimen of 6 hour/day on 5 consecutive exposure days/week. As can be deduced from Figure 7, findings from short-term high-dose studies are biased towards MoA_I_-related outcomes with surface area/activity as lead metric whereas long(er)-term studies are better suited of minimizing the relative contribution of the MoA_I_ (Figure 6), ideally supported by physiology-based kinetic modeling [36].

### Simulated study design and verification of prediction

**Figure 8:**
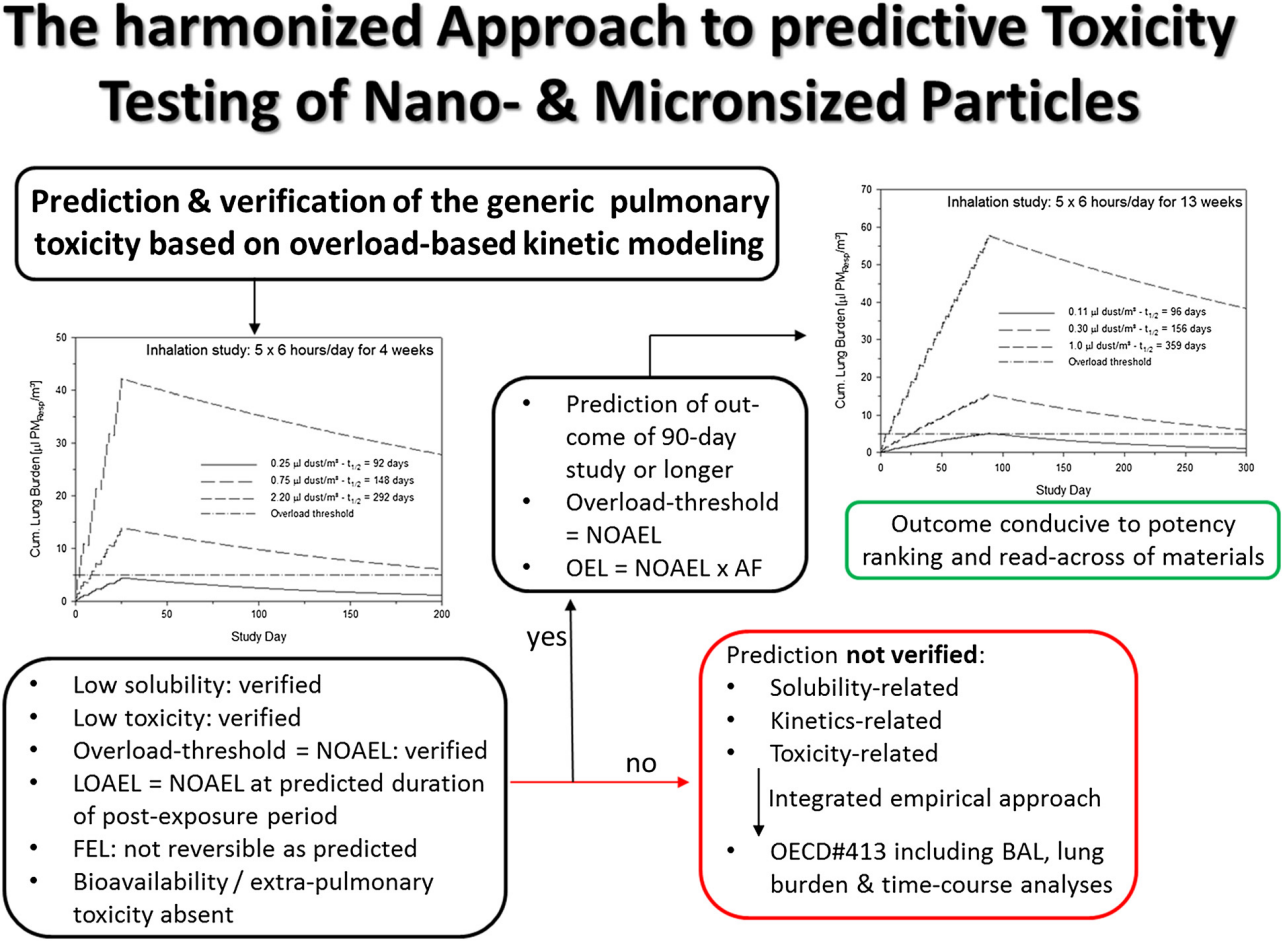
**Kinetically simulated 4-week (left) and 13-week (right) OECD#412/413** **[** 8 **-** 10 **]** **compliant inhalation studies on rats for estimation of the volume concentration required to attain a cumulative PSP-volume lung burden similar to the NOAEL and the MTD.** Key is the selection of an intermediate concentration to demonstrate reversible lung-overload and associated adverse outcomes [4,36] within the kinetically simulated post-exposure period. Simulations used volumetric concentrations of 0.25, 0.75, and 2.2 μl PSP_resp_/m^3^ for the 4-week study and of 0.11, 0.3, and 1.0 μl PSP_resp_/m^3^ for the 13-week study. The simulations rely upon the correct estimate of that MPPD-modeled respirability and agglomerate density achieved in the particular study under consideration (for details see [4,6,23]). Any faster reversibility than predicted would have suggested facilitated dissolution of PSP in the lung. In case all constraints of the 4-week inhalation study are fulfilled, the NOAEL and OEL/DNEL from longer exposure periods can be predicted. Adjustments for exposure durations (AF) have been detailed elsewhere [4,5]. Modelling is not supported for substances where the predicted outcome of the 4-week study does not match the empirical outcome.

The wealth of data from biopersistent, low-toxicity PSP repeated exposure inhalation studies on rats support a common toxic principle [4]. Due to the low toxicity of typical PSP, the comparison of many of these studies is often hampered by differences in study design, lack of lung burden analyses and fate, unexpected solubility, and post-exposure periods shorter than the macrophage mediated half-time of 60 days. The study design depicted in Figure 8 attempts to minimize these types of data gaps by demonstrating the coherence of the kinetically predicted and empirically verified cumulative lung dose with any integrated pulmonary effect. Based on the details given in Figures 5, 6 and 7, the lung burden-related cornerstones of any repeated inhalation study can be simulated (for details see [4]). The key study is then restricted to a fully simulated study embracing the NOAEL to the MTD and a kinetically calculated post-exposure period suitable to demonstrate full reversibility at the intermediate dose with no reversibility at the high-level dose (1^st^ step in Figure 8). Such a study is commonly preceded by a validating, exploratory 1-week study for technical optimization and validation. Few animals exposed at this exposure level serve the purpose to validate the analytical determination of lung burdens observing post-exposure periods long enough to detect retention half-times shorter than t_1/2_ = 60 days. In case this criterion is fulfilled, the substance under consideration may require additional qualification tests.

For a proven PSP, the targeted cornerstones of study can be modeled in a 4-week OECD#412 [8,9] compliant inhalation study using volumetric concentrations of 0.25, 0.75, and 2.2 μl PSP_resp_/m^3^. The respirable fraction of PSP (PSP_resp_) is that defined by OECD-GD#39 (2009). The predicted elimination half-times are to be expected in the range of 92, 148, and 292 days, respectively. Confidence limits are not given to these point estimates due to the numerous methodological variables affecting ‘inhaled and retained dose’. Therefore, these and other estimates from simulations need to be put into context with the confidence intervals of the respective PODs from the modeled and empirically verified repeated 4-week exposure inhalation study. As exemplified elsewhere [23], for entirely new and complex structures (Multi-walled Carbon nanotubes) a modeled and empirically verified 13-week OECD#413 [8,10] seems to be the more appropriate and conservative approach. Mass concentrations are calculated by multiplication of the apparent density of PSP with the volume concentration. In case the empirical outcome matches the prediction (Figure 8), the NOAELs from longer study derations can then be predicted with reasonable accuracy [4,36]. In case an entirely novel type of PSP with yet unknown toxicological properties is tested, the same cycle using a 13-week exposure period may be considered. The OEL can be derived based on the calculated and experimentally verified NOAEL using an assessment factor (AF) to adjust the actual study duration to chronic exposure as conceptualized in Figure 7. In the absence of kinetically designed studies and empirically confirmed lung burdens and dosimetry substance-specific and retained-dose-related effects cannot be unequivocally differentiated [46].

### Mechanisms, human relevance, and impact on risk assessment

Employers are legally obliged to provide a working environment that does not endanger the health of employees (e.g. Chemical Agent Directive 98/24/EC and Framework Directive 89/391/EEC) [2,3]. Occupational exposure limits (OELs) or Derived Human No-Effect Levels (DNELs) are a useful instrument for the prevention of health effects during the handling and use of chemical substances [1]. OELs are defined as airborne concentrations (expressed as time-weighted average for a conventional 8-hour work day and a 40-hour work week) of a substance to which it is believed that nearly all workers may be repeatedly exposed (day after day, for a working lifetime) without adverse effect. OELs are derived in context with the registration and/or notification of chemicals. Those derived by multi-expert groups (e.g., ACGIH, DFG, SCOEL [47-49]) become legally binding workplace standards. Exposure concentrations are expressed in mg/m^3^.

Dosimetry adjustments across species of particles deposited in the various regions of the respiratory tract and especially the alveoli require critical mode of action (MoA)-based (dynamic) and kinetic information interrelating dynamics with biopersistence (mediated by AM clearance with negligible influence of dissolution). Because insoluble particles deposit, accumulate and clear, *inter alia*, along the surface of the lower respiratory tract, normalization factors may differ from one MoA and accumulating compartment to another [12]. Published evidence suggests that the adaptively increase in the pooled volume of alveolar phagocytes (Vd) matches the increased payload of lung burdens best (MoA_II_) [4]. The NOAEL from kinetically modeled and experimentally verified rat repeated inhalation studies (Figure 8) is considered to be the POD for OEL-derivation in the absence of additional species-specific susceptibility factors [7]. Notably, based on the concept to prevent ‘kinetic overload’ and associated inflammation, the volume-based generic mass concentration of 0.54 μl PSP_resp_ × ρ/m^3^ (ρ = apparent density of the PSP accumulated in the phagocyte) appears to be scientifically justified and defensible as a generic OEL for preventing PSP-induced pulmonary overload-like conditions to occur in chronically exposed workers [4-6].

Mammalian species have developed finely-tuned lungs adapted to their size and physiological needs. These must be operational from a 25 g mouse with 300 breaths/minute to a 70 kg human with 15 breaths/minute. The basic structures involving PSP clearance and surfactant homeostasis, have remarkable consistency across species [35]. As can be expected, complex physiological relationships exist between alveolar size and surface forces and the way they coexist to maintain alveoli in a stable configuration. This interrelationship follows from the Laplace relation (intra-alveolar pressure = 2 × surface tension/alveolar radius) showing that alveolar stability requires that smaller alveoli of smaller mammalian species have more functional surfactant than larger alveoli [50]. This intricate balance is further complicated because smaller lungs and airways to not allow for large reservoirs accommodating surfactant and, if out of tune, mucus-like substances might compromise lung function by the plugging small airways. This particular configuration makes the lung of small laboratory rodents ultimately susceptible to high doses of solid materials capable to adsorb and/or deteriorate pulmonary surfactant.

Many mechanisms readily operative in humans to buffer away such mechanical stimuli may not be feasible in rodents. These types of MoA_I_-related etiopathologies need to be thoughtfully characterized and put into human perspective for a balanced risk characterization. It has been shown that the alveolar macrophage from humans is larger than that from rats [51]. In addition, also the total number of alveolar macrophages in the lungs of humans is reported to be higher in humans as compared to rats [4,21,52]. This circumstance makes humans about 7-times more resistant to attaining lung overload-like conditions than rats. Keeping in mind that the area patrolled by each AM can change dramatically from one species to another, e.g, rat with 140,000 μm^2^/AM vs. human 22,000 μm^2^/AM [52] humans seem to be capable to keep their lung surface clean with a 7-fold higher capacity as compared to rats which is entirely coherent with pool volume-based estimate of species differences. Likewise, it is also important to recall that the alveolar region of the rat and human has a lining fluid (LF) volume of 26.4 and 8900 μl, respectively [53]. Accounting for a pulmonary surface area of 0.34 m^2^ [54] and 54 m^2^ [55] this then results in 78 and 165 μl LF/m^2^ for rats and humans, respectively. These considerations support the concept that there is a much higher intrinsic susceptibility of lungs of rats’ as compared to humans.

At occupational settings this type of portal-of-entry related toxicity (as a result of particle overload) is independent on any local metabolism. It also appears, as if the cornerstones of particle clearance appear to be well conserved in mammalian species and do not call for any specific rat-to-human adjustments [35]. Therefore, these bioassay-specific ‘implicit adjustment factors’ advocate to focus on dosimetric species differences only (inhaled volume per exposure day, respirability of particles, and differences in elimination kinetics) as already conceptualized elsewhere [4]. Related to mass concentrations any OEL of any proven typical PSP can readily be calculated by multiplication of the volume concentration with the PM-agglomerate density (ρ): OEL or DNEL [mg/m^3^] = 0.54 μl PM_resp_/m^3^ × ρ; ρ = 1 g/cm^3^). Interestingly, the conclusions of this paper match almost exactly the deliberations of independent, multi-disciplinary expert groups engaged with the derivation of legally binding occupational exposure metric and limits of PSP (DFG-MAK Commission, Committee on Hazardous Substances): MAK_DFG_ [mg/m^3^] = 0.3 μl PM_resp_/m^3^ × ρ [56,57], TRGS900-value = 0.5 μl PM_resp_/m^3^ × ρ [58] (both limits adjusted to mass/m^3^ using an apparent density of ρ = 1g/cm^3^). Thus, similar to the approach described in this paper, also the general dust limit value considers the overload-dependent PSP-volume metric to be superior to any elusive surface area related metric as already suggested previously [3,36]. As typical for these types of values, uncertainty is considered by implicit assessment factors rather than any statistically derived confidence limits [12].

## Summary and conclusions

This treatise demonstrates that essentially two independent modes of action may act in concert in PSP-related pulmonary toxicity. These are dependent on the cumulative dose and regimen chosen to characterize hazards of PSP. Due to its link to chronic workplace exposure, MoA_II_ retention-related effects are considered to be amongst the key event leading to overload-dependent pulmonary inflammation which occurrence must be prevented with certainty. In using kinetically-modeled 4-week inhalation studies on rats (Figure 8) any presumed PSP can unequivocally be classified as typical or non-typical PSP in terms of dissolution kinetic and AM-mediated clearance kinetic or any other yet unaccounted toxicity. Accumulated empirical evidence from chronic inhalation studies with true PSP yielded NOAELs similar to the PSP-specific NOAEL or the predicted generic NOAEL [4,6]. However, caution is advised in using these paradigms for novel types of PSPs with unusual surface thermodynamics and chemistry, complex kinetics of dissolution and/or yet uncharacterized PSP-specific toxicity. Hypothesis-based kinetically-designed repeated inhalation studies with fewer animals will minimize resource consuming inhalation studies with outcomes determined by PSP-specific rather than overload-related unspecific effects. This provides a solid basis for a better comparison of PSPs with different characteristics as well as prediction of safe occupational exposure levels in the future.

## References

[1] ECETOC (European Centre for Ecotoxicology and Toxicology of Chemicals). Guidance on Assessment Factors to Derive a DNEL. Technical Report No. 110, November 2010. Available at: http://members.ecetoc.org/Documents/Document/20110131112906-ECETOC_Technical_Report_110.pdf

[2] EC (1998). Council Directive 98/24/EC of 7 April 1998 on the protection of the health and safety of workers from the risks related to chemical agents at work (fourteenth individual Directive within the meaning of Article 16(1) of Directive 89/391/EEC. Off J Eur Comm.

[3] ECHA, 2008. Guidance on Information Requirements and Chemical Safety Assessment, Chapter R. 8: Characterisation of Dose [Concentration]-Response for Human Health. European Chemicals Agency (ECHA), Available at: http://echa.europa.eu/documents/10162/13632/information_requirements_r8_en.pdf

[4] Pauluhn J (2011). Poorly soluble particulates: searching for a unifying denominator of nanoparticles and fine particles for DNEL estimation. Toxicology.

[5] Pauluhn J (2010). Multi-walled carbon nanotubes (Baytubes®): approach for derivation of occupational exposure limit. Regul Toxicol Pharmacol.

[6] Pauluhn J: **Subchronic inhalation toxicity of iron oxide (magnetite Fe**_**3**_**O**_**4**_**) in rats: toxic effects are determined by the particle kinetics typical of poorly soluble particles not particle dynamics.** *J Appl Toxicol* 2012, **32:**488–504. 10.1002/jat.1668 21456093

[7] ECETOC (European Centre for Ecotoxicology and Toxicology of Chemicals) Poorly Soluble Particles / Lung Overload, Technical Report 122, 20 January 2014. Available at: http://www.ecetoc.org/index.php?mact=Newsroom,cntnt01,details,0&cntnt01documentid=237&cntnt01returnid=76

[8] OECD Environment, Health and Safety Publications, Series on Testing and Assessment No. 39: Guidance Document for Acute Inhalation Toxicity Testing, July 21, 2009; Available at: http://ntp.niehs.nih.gov/iccvam/suppdocs/feddocs/oecd/oecd-gd39.pdf

[9] OECD Guidelines for the Testing of Chemicals, Section 4: Test No. 412: Subacute Inhalation Toxicity: 28-Day Study, 8 Sept 2009. Available at: http://www.oecd-ilibrary.org/docserver/download/9741201e.pdf?expires=1415622962&id=id&accname=guest&checksum=DEEA3D8F9D98773360AC8279D38F9D48

[10] OECD Guidelines for the Testing of Chemicals, Section 4: Test No. 413: Subchronic Inhalation Toxicity: 90-day Study, 8 Sept 2009. Available at: http://www.oecd-ilibrary.org/environment/test-no-413-subchronic-inhalation-toxicity-90-day-study_9789264070806-en

[11] UNECE (United Nations Economic Commission for Europe): Globally Harmonized System of Classification and Labelling of Chemicals (GHS), Fifth revised edition, Part 3: Health Hazards. GHS (Rev.5), 2013. Available at: http://www.unece.org/ru/trans/danger/publi/ghs/ghs_rev05/05files_r.html

[12] U.S.-EPA. Methods for derivation of inhalation reference concentrations and application of inhalation dosimetry. EPA/600/8-90/066F, October 1994. Available at: http://cfpub.epa.gov/ncea/cfm/recordisplay.cfm?deid=71993

[13] OECD: Environment Directorate - Joint Meeting of the Chemicals Committee and the Working Party on Chemicals Pesticides and Biotechnology Important Issues on Risk Assessment of Manufactured Nanomaterials, *Series on the Safety of Manufactured Nanomaterials* No. 33 (28 March 2012) Available from: http://search.oecd.org/officialdocuments/displaydocumentpdf/?cote=env/jm/mono%282012%298&doclanguage=en

[14] Morrow PE (1992). Dust overloading in the lungs. Toxicol Appl Toxicol.

[15] Morrow PE (1988). Possible mechanisms to explain dust overloading of the lungs. Fundam Appl Toxicol.

[16] Morrow PE: Mechanisms and significance of “particle overload”. In *Toxic and carcinogenic effects of solid particles in the respiratory tract: [Proceedings of the 4*^*th*^*international inhalation symposium]* March 1993, Hannover Germany 1994, pp. 17–25 Washington DC: International Life Sciences Institute press.

[17] YU CP, Chen YK, Morrow PE (1989). Analysis of alveolar macrophage mobility kinetics at dust overloading of the lungs. Fundam Appl Toxicol.

[18] Oberdoerster G (2002). Toxicokinetics and effects of fibrous and nonfibrous particles. Inhal Toxicol.

[19] Oberdoerster G, Oberdoerster E, Oberdoerster J (2007). Concepts of nanoparticle dose metric and response metric. Environ Health Perspect.

[20] Oberdoerster G, Ferin J, Morrow PE (1992). Volumetric loading of alveolar macrophages (AM): a possible basis for diminished AM-mediated particle clearance. Exp Lung Res.

[21] Oberdoerster G (1995). Lung particle overload: Implications for occupational exposures to particles. Regul Toxicol Pharmacol.

[22] Pauluhn J (2014). Repeated inhalation exposure of rats to an anionic high molecular weight polymer aerosol: application of prediction models to better understand pulmonary effects and modes of action. Exp Toxicol Pathol.

[23] Pauluhn J (2010). Subchronic 13-week inhalation exposure of rats to multi-walled carbon nanotubes: toxic effects are determined by density of agglomerate structures not fibrillar structures. Toxicol Sci.

[24] OECD, 2013. Environment Directorate – Joint meeting of the chemicals committee and the working party on chemicals, pesticides, and biotechnology: Guidance Document on the Developing and Assessing Adverse Outcome Pathways, Series on Testing and Assessment No. 184. (ENV/JM/MONO(2013)6 as of April 17, 2013). Available at http://www.oecd.org/officialdocuments/publicdisplaydocumentpdf/?cote=env/jm/mono%282013%296&doclanguage=en

[25] Brunner L, Tolloczko S (1900). Über die Auflösungsgeschwindigkeit Fester Körper. Zeitschrift für Physikalische Chemie.

[26] Brunner E (1900). Reaktionsgeschwindigkeit in heterogenen Systemen. Zeitschrift für Physikalische Chemie.

[27] Nernst W (1904). Theorie der Reaktionsgeschwindigkeit in heterogenen Systemen. Zeitschrift für Physikalische Chemie.

[28] Dokoumetzidis A, Macheras P (2006). A century of dissolution research: from Noyes and Whitney to the Biopharmaceutics classification system. Int J Pharmaceutics.

[29] Brittain HG (2014). Thermodynamic vs.

[30] Wong ST (2007). Computer-Aided Modeling of Controlled Release Through Surface Erosion With and Without Microencapsulation. Graduate Theses and Dissertations.

[31] Pauluhn J, Rosenbruch M (2003). Inhalation toxicity of propineb part I: results of subacute inhalation exposure studies in rats. Inhal Toxicol.

[32] Pauluhn J, Emura M, Mohr U, Rosenbruch M (2003). Inhalation toxicity of propineb part II: results of mechanistic studies in rats. Inhal Toxicol.

[33] Ho M, Wu KY, Chein HM, Chen LC, Cheng TJ (2011). Pulmonary toxicity of inhaled nanoscale and fine zinc oxide particles: mass and surface area as an exposure metric. Inhal Toxicol.

[34] Adamcakova-Dodd A, Stebounova LV, Kim JS, Vorrink SU, Ault AP, O’Shaughnessy PT, Grassian VH, Thorne PS (2014). Toxicity assessment of zinc oxide nanoparticles using sub-acute and sub-chronic murine inhalation models. Part Fibre Toxicol.

[35] Wirkes A, Jung K, Ochs M, Mühlfeld C (2010). Allometry of the mammalian intracellular pulmonary surfactant system. J Appl Physiol.

[36] Pauluhn J (2014). The metrics of MWCNT-induced pulmonary inflammation are dependent on the selected testing regimen. Reg Pharmacol Toxicol.

[37] Brown JS, Wilson WE, Grant LD (2005). Dosimetric comparisons of particle deposition and retention in rats and humans. Inhal Toxicol.

[38] US-EPA (US Environmental Protection Agency): Toxicological Review of Barium and Compounds. Washington DC 1998. (available at: http://www.epa.gov/iris/toxreviews/0010tr.pdf)

[39] Brunauer S, Emmet PH, Teller E (1938). Adsorption of gases in multimolecular layers. J Am Chem Soc.

[40] Landsiedel R, Ma-Hock L, Hofmann T, Wiemann M, Strauss V, Treumann S, Wohlleben W, Gröters S, Wiench K, van Ravenzwaay B (2014). Application of short-term inhalation studies to assess the inhalation toxicity of nanomaterials. Part Fibre Toxicol.

[41] Cullen RT, Tran CL, Buchanan D, Davis JM, Searl A, Jones AD, Donaldson K (2000). Inhalation of poorly soluble particles I: differences in inflammatory response and clearance during exposure. Inhal Toxicol.

[42] Tran CL, Buchanan D, Cullen RT, Searl A, Jones AD, Donaldson K (2000). Inhalation of poorly soluble particles II: Influence of particle surface area on inflammation and clearance. Inhal Tox.

[43] ICRP (International Commission on Radiological Protection): Annals of the ICRP - Draft Report for Consultation - Occupational Intakes of Radionuclides Part 1. *ICRP* ref 4828-2081-0510 February 23, 2012. Available at: http://www.icrp.org/page.asp?id=155.

[44] Stöber W, McClellan RO (1997). Pulmonary retention and clearance of inhaled biopersistent aerosol particles: data-reducing interpolation models and models of physiologically based systems. A review of recent progress and remaining problems. Crit Rev Toxicol.

[45] Maynard AD (2007). Nanotechnology: the next big thing or much ado about nothing?. Ann Occup Hyg.

[46] Pauluhn J, Rosenbruch M: **Lung burdens and kinetics of multi-walled carbon nanotubes (Baytubes) are highly dependent on the disaggregation of aerosolized MWCNT.** *Nanotoxicology* 2014, **19:**1–11. [Epub ahead of print]. 10.3109/17435390.2014.918204 24842705

[47] ACGIH: TLVs and BEIs. Threshold limit values for chemical substances and physical agents. American Conference of Governmental and Industrial Hygienists, Cincinnati, OH, USA, 2014. Available at: www.acgih.org/Products/tlvintro.htm.

[48] DFG: The MAK Collection for Occupational Health and Safety, 2014. Available online at: (gerneral); http://onlinelibrary.wiley.com/book/10.1002/3527600418/topics (technical documents/methods); http://onlinelibrary.wiley.com/book/10.1002/9783527675135 (MAK- and BAT-values).

[49] SCOEL: *The Scientific Committee on Occupational Exposure Limits.* Links available at: http://en.wikipedia.org/wiki/Scientific_Committee_on_Occupational_Exposure_Limit_Values.

[50] Lum H, Mitzner W (1987). A species comparison of alveolar size and surface forces. J Appl Physiol.

[51] Krombach F, Münzing S, Allmeling AM, Gerlach JT, Behr J, Dörger M (1997). Cell size of alveolar macrophages: an interspecies comparison. Environ Health Perspect.

[52] Valberg PA, Blanchard JD: Pulmonary Macrophage Physiology: Origin Motility Endocytosis *In Treatise on Pulmonary Toxicology – Comparative Biology of the normal Lung* (editor Richard A Parent), CRC Press 1992, Boca Raton pp. 681–724

[53] Hatch GE: Comparative Biochemistry of Airway Lining Fluid *In Treatise on Pulmonary Toxicology – Comparative Biology of the normal Lung* (editor Richard A Parent), CRC Press 1992, Boca Raton pp. 617–632

[54] Gehr P, Mwangi DK, Ammann A, Taylor CR, Weibel ER (1981). Design of the mammalian respiratory system V Scaling morphometric pulmonary diffusing capacity to body mass: wild and domestic mammals. Respir Physiol.

[55] Mercer RR, Russel ML, Crapo JD (1994). Alveolar septal structure in different species. J Appl Physiol.

[56] DFG: The MAK Collection for Occupational Health and Safety, General threshold limit value for dust (R fraction and biopersistent granular dust), 2011. Available online at: http://onlinelibrary.wiley.com/doi/10.1002/3527600418.mb0230stwd0053/pdf

[57] BAuA: (Federal Institute for Occupational Safety and Health), AGS public hearing: “A New General threshold limit value for dust”, March 2013. Available at: http://www.baua.de/de/Themen-von-A-Z/Gefahrstoffe/AGS/pdf/AGS-publik-2013-2.pdf?

[58] TRGS 900 (2014). Begründung zum Allgemeinen Staubgrenzwert (2014/2001). Bekanntmachung von Technischen Regeln hier: − TRGS 900, Arbeitsplatzgrenzwerte.

